# The Pharmaceutical Society

**Published:** 1915-02-20

**Authors:** 


### THE PHARMACEUTICAL SOCIETY.

It is apparent from the report of the Registrar of the Pharmaceutical Society which has just been issued that there is no falling off in the number of qualified pharmacists. The number of names on the present register is, in fact, somewhat larger as compared with last year's register, and although pharmacy has contributed its quota to the new army, it is satisfactory to note a material increase in the number of apprentices who will be presenting themselves for the qualifying examination in due course. To be precise, the number of names on the Pharmaceutical Register is 16,630, as against 16,608 in the previous year, while the number of registered apprentices is about 20 per cent. larger than it was a year ago. No doubt the improved conditions of the pharmaceutical business are responsible for attracting more students; during the last year or two the salaries obtainable by young chemists employed as dispensers in hospitals and as assistants in pharmacies have risen, while the outlook for those who practise on their own account seems to have become brighter. The membership of the Society continues to increase, and is now 8,175, the largest it has ever been, and nearly two thousand more than it was ten or eleven years ago. Quite half of those who are eligible for membership are now subscribers to the Society. For a voluntary membership this is satisfactory, especially in view of the fact that the membership comprises most of those who have pharmacies of their own.

The administration of the penal clauses of the Pharmacy Acts, which is one of the Society's most important duties, continues to entail a vast amount of work. This is evident from the fact that last year over a thousand cases of alleged infringement of these Acts had to be investigated; proceedings were instituted in 239 cases. In the majority of instances the irregularities consisted in the selling of poisons by unqualified persons—a practice which, in the interests of the public, should be prevented as far as possible.

